# Antibacterial and Antibiofilm Activity of Iron Oxide Nanoparticles: An Integrative Systematic Review

**DOI:** 10.3390/antibiotics15101014

**Published:** 2026-10-13

**Authors:** Jessica Alves dos Santos, Adriana Bozzi, Marianna Gabrielle Almeida Galvão, Aline Silva

**Affiliations:** 1 Programa de Pós-Graduação Biologia e Biotecnologia de Microrganismos (PPGBBM), Universidade Estadual de Santa Cruz, Ilhéus 45662-900, BA, Brazil; jessica.edionemedson@hotmail.com (J.A.d.S.); marianna.galvao@hotmail.com (M.G.A.G.); 2 Department of Biological Sciences, Universidade Estadual de Santa Cruz, Ilhéus 45662-900, BA, Brazil; abozzi@uesc.br

**Keywords:** iron oxide nanoparticles, antimicrobial resistance, biofilm inhibition, Gram-positive bacteria, Gram-negative bacteria, nanoparticle and antibiotic synergy, multidrug resistant bacteria

## Abstract

**Background/Objectives**: The global rise in antimicrobial resistance has intensified the search for alternative therapeutic strategies capable of overcoming bacterial tolerance and biofilm-associated infections. Iron oxide nanoparticles (IONPs) have emerged as promising candidates due to their physicochemical properties and potential multimodal mechanisms of action. This integrative review aimed to evaluate the antibacterial and antibiofilm activity of iron oxide nanoparticles against Gram-positive and Gram-negative bacteria, as well as their potential synergistic effects with conventional antibiotics. **Methods**: A structured search was conducted in PubMed, Web of Science, BVS, LILACS, and Redalyc databases using predefined descriptors related to iron nanoparticles and bacterial inhibition. Fifteen studies published between 2014 and 2024 met the inclusion criteria. The methodological quality of the included studies was assessed using the Joanna Briggs Institute (JBI) Critical Appraisal Checklist. Most studies presented low to moderate risk of bias and the findings were categorized into three thematic areas: antibacterial activity of IONPs, synergistic interaction with antibiotics, and reported adverse effects. **Results**: Overall, IONPs demonstrated inhibitory effects against clinically relevant pathogens, including *Escherichia coli*, *Staphylococcus aureus*, and *Pseudomonas aeruginosa*, showing activity against both planktonic cells and biofilm formation. Synergistic effects with antibiotics were frequently reported, suggesting enhanced antimicrobial efficacy potentially mediated by increased membrane permeability and reactive oxygen species generation. **Conclusions**: Iron oxide nanoparticles show promising antibacterial and antibiofilm effects and may act as adjuncts to conventional antibiotics. Nevertheless, further standardized and translational studies are required before clinical implementation.

## 1. Introduction

Nanotechnology has emerged as a rapidly expanding field with applications across medicine, biotechnology, and materials science. Among nanomaterials, iron oxide nanoparticles (IONPs), typically defined as particles smaller than 100 nm, exhibit unique optical, thermal, magnetic, and electrochemical properties that support their wide applicability in biomedical research [1,2]. These physicochemical characteristics have enabled the investigation of IONPs in diverse fields, including antimicrobial therapy and the control of microbial growth.

Several types of nanoparticles demonstrate antimicrobial activity, including silver (Ag), zinc oxide (ZnO), titanium dioxide (TiO_2_), gold (Au), silicon (Si), and iron oxide nanoparticles (IONPs) [3,4]. Among these, IONPs are particularly attractive due to their relative biocompatibility, magnetic properties, and potential multifunctionality. In healthcare, IONPs have been explored as drug delivery systems and antimicrobial compounds, either alone or in combination with conventional antibiotics, allowing targeted and controlled release while potentially reducing systemic toxicity [2,3].

Experimental studies have demonstrated that IONPs exhibit antimicrobial activity against a broad spectrum of pathogens, including Gram-positive bacteria such as *Staphylococcus aureus* and *Bacillus nealsonii*, and Gram-negative bacteria such as *Delftia acidovorans* and *Escherichia coli* [4,5]. However, susceptibility is not uniform, and some microorganisms demonstrate reduced sensitivity, highlighting the need for comparative microbiological evaluation.

The antimicrobial effects of IONPs are influenced by several parameters, including synthesis method, particle size, morphology, surface functionalization, and concentration. Proposed mechanisms of action include disruption of bacterial cell membranes, interference with efflux pumps, inhibition of resistance-associated genes, DNA damage, and generation of reactive oxygen species (ROS), which play a central role in oxidative stress-mediated bacterial killing [6,7].

The urgency of investigating alternative antimicrobial strategies is reinforced by the global rise in antimicrobial resistance (AMR). The inappropriate and excessive use of antibiotics has accelerated the emergence of multidrug-resistant pathogens, compromising the effectiveness of current therapies [8]. In 2019, major bacterial pathogens such as *Escherichia coli*, *Staphylococcus aureus*, *Klebsiella pneumoniae*, *Streptococcus pneumoniae*, *Acinetobacter baumannii*, and *Pseudomonas aeruginosa* were responsible for a substantial proportion of AMR-associated mortality worldwide [9]. Increasing resistance to fluoroquinolones, third-generation cephalosporins, methicillin, and even last-resort agents such as vancomycin underscores the pressing need for novel therapeutic approaches [10].

Antimicrobial resistance (AMR) represents a critical and escalating global public health threat. A global analysis published in *The Lancet* estimated that bacterial AMR was directly responsible for 1.27 million deaths and contributed to 4.95 million deaths worldwide in 2019 [9]. Surveillance reports from the World Health Organization (WHO) document high prevalence rates of resistance in common pathogens such as *Escherichia coli* and *Klebsiella pneumoniae* to widely used antibiotics, with the most significant burdens observed in low- and middle-income countries [11]. Historical projections underscore the potential magnitude of this crisis, suggesting that resistant infections could cause up to 10 million annual deaths by 2050 in the absence of effective interventions, with profound implications for morbidity, mortality, treatment failure, and healthcare costs [12].

This context necessitates the exploration of complementary strategies to conventional antibiotics, and nanotechnology-based approaches have emerged as a promising avenue, offering the potential to enhance drug pharmacokinetics, increase local concentration at the site of infection, and potentially restore antibiotic efficacy against resistant strains [13]. Among these, iron oxide nanoparticles have attracted significant research interest due to their antibacterial and antibiofilm properties. Additionally, iron oxide nanoparticles have been integrated into photodynamic platforms, where superparamagnetic cores coated with photosensitizers such as methylene blue can be magnetically guided and activated by red light to generate bactericidal ROS against *E. coli* and other pathogens [14].

In addition to planktonic resistance, biofilm formation represents a major challenge in clinical settings, as biofilm-embedded bacteria exhibit significantly enhanced tolerance to antimicrobial agents and host immune responses. Therefore, strategies capable of targeting both bacterial growth and biofilm formation are of particular interest.

Despite the growing number of experimental studies evaluating iron oxide nanoparticles as antimicrobial agents, the available findings remain heterogeneous and fragmented due to differences in nanoparticle synthesis routes, physicochemical characterization, bacterial strains tested, and experimental methodologies including planktonic and biofilm models [15,16]. In addition, most existing reviews address nanomaterials or iron oxide nanoparticles in a broad biomedical context, and a focused synthesis that comparatively examines antibacterial and antibiofilm activity, while also considering synergistic effects with antibiotics, is still limited.

Therefore, this integrative review aims to systematically evaluate the antibacterial and antibiofilm activity of iron oxide nanoparticles by (i) analyzing their effects against Gram-positive and Gram-negative bacteria, (ii) exploring their synergistic interactions with conventional antibiotics, and (iii) relating these outcomes to microbiological mechanisms and nanoparticle characteristics, in order to provide understanding of their antimicrobial potential.

## 2. Results

The study selection process is summarized in Figure 1. A total of 688 records were identified across all selected databases during the initial search. After removal of duplicates and application of the predefined inclusion and exclusion criteria, 15 studies were considered eligible and included in the qualitative synthesis.

The main characteristics of the included studies are summarized in Table 1, including authorship, nanoparticle type, bacterial species and Gram (G) classification, particle size, bacterial inhibition (MIC), biofilm effects and synergy with antibiotics.

The methodological quality assessment indicated that most studies presented low risk of bias, particularly regarding control groups and antimicrobial outcome measurements. Moderate risk classifications were primarily associated with incomplete reporting of nanoparticle physicochemical characterization. No study was classified as high risk of bias (Table 2).

## 3. Discussion

### 3.1. Use of Iron Nanoparticles as Antibacterial Agents

The studies included in this review demonstrate that iron oxide nanoparticles (IONPs) exhibit antimicrobial activity against a broad spectrum of Gram-positive and Gram-negative bacteria. However, antibacterial efficacy varied depending on the nanoparticle concentration, physicochemical characteristics, and bacterial species evaluated [15,20,22,26,28].

Most investigations reported concentration-dependent effects, with inhibitory activity observed within microgram to milligram ranges, reflecting differences in nanoparticle formulation and experimental design [15,20,26]. In the study by Gabrielyan et al. [15], concentration-dependent growth inhibition was clearly demonstrated, with bactericidal effects observed at higher concentrations. Similarly, Fatih et al. [28] reported significant antibacterial and antibiofilm activity of green-synthesized IONPs against pathogenic bacteria.

The variability in antimicrobial activity appears strongly associated with nanoparticle size, surface modification, synthesis method, and aggregation behavior [26]. Surface-functionalized nanoparticles demonstrated enhanced antibacterial performance, possibly due to improved interaction with bacterial membranes and increased ROS production [26,28]. Although Gram-negative bacteria possess an outer membrane that may restrict nanoparticle penetration, studies demonstrated inhibitory activity against species such as *Escherichia coli* and *Pseudomonas aeruginosa* [15,28].

Regarding antibiofilm activity, Pham et al. [18] demonstrated that nanoparticles inhibited biofilm formation and modulated virulence and motility properties in *P. aeruginosa*. Likewise, Armijo et al. [21] observed antibiofilm effects when iron-based nanoparticles were tested against *P. aeruginosa* biofilms. These findings are particularly relevant since biofilm-associated bacteria exhibit increased tolerance to antibiotics.

In addition to planktonic models, iron oxide-based coatings have also been evaluated directly on the surface of clinically relevant medical devices, demonstrating that iron nanoparticles functionalized with Cinnamomum verum and deposited on gastrostomy tubes significantly reduced the colonization and biofilm formation of both *S. aureus* and *E. coli*, including mature biofilms, while maintaining the functional integrity of the device surface. These findings reinforce the potential of IONP-based coatings as antibiofilm strategies for the prevention of device-associated infections [16].

Mechanistically, antibacterial effects were associated with ROS generation, membrane disruption, and interference with cellular integrity [15,29]. The iron may enhance oxidative stress, contributing to bacterial cell damage [15]. These mechanistic pathways are summarized schematically in Figure 2.

In the broader context of metal-based nanomaterials, iron oxide nanoparticles appear to display more moderate intrinsic antibacterial activity when compared with some other metal nanoparticles, such as silver or zinc oxide, which are frequently reported as strongly bactericidal at lower concentrations but may also raise greater concerns regarding cytotoxicity and environmental impact [3,5,6]. In contrast, IONPs are generally regarded as relatively biocompatible and offer additional functionalities, including magnetic responsiveness and the possibility of targeted delivery, which may be advantageous for their use as adjuncts to conventional antimicrobials rather than as standalone bactericidal agents [1,2,3,7].

### 3.2. Evaluation of Iron Nanoparticles in Synergy with Antibiotics

An important finding of this review is the synergistic interaction reported between IONPs and conventional antibiotics. In several studies, nanoparticle–antibiotic combinations demonstrated greater antibacterial efficacy compared to antibiotics alone [13,21,23,27].

De Sousa et al. [27] evaluated rifampicin associated with iron oxide nanoparticles and observed enhanced antimicrobial performance. Similarly, Armijo et al. [21] reported improved antibiofilm activity when iron-based nanoparticles were conjugated with tobramycin. Shahbazi et al. [19] demonstrated bacteriostatic effects of iron oxide nanoparticles in synergy with antibiotics against *Enterococcus faecalis*, while Abdulsada et al. [23] reported increased antibacterial activity of iron oxide nanoparticles conjugated with gentamicin against multidrug-resistant strains. In addition to aminoglycosides and rifampicin, IONPs have also been used to enhance the efficacy of β-lactam antibiotics. Biocompatible IONPs improved amoxicillin delivery and significantly increased its antibacterial activity against both *S. aureus* and *E. coli*, compared with the free drug. These findings further support the role of iron oxide nanoparticles as versatile adjuvants capable of potentiating different antibiotic classes [17].

Fimbriae-targeted superparamagnetic iron oxide nanoparticles loaded with ciprofloxacin achieved ~97% killing of quinolone-resistant *E. coli* and up to 98.5% biofilm inhibition at ciprofloxacin concentrations 8–16 times lower than the MBEC of the free antibiotic, underscoring the potential of IONP-based systems to restore antibiotic efficacy against resistant biofilm-forming strains [24].

These synergistic effects may be attributed to increased membrane permeability, facilitation of antibiotic internalization, and oxidative stress enhancement [15,27]. Such findings are particularly relevant in the context of antimicrobial resistance, as nanoparticle-based strategies may reduce the required antibiotic dose and potentially restore susceptibility in resistant bacteria.

However, the degree of synergy varied among studies, and standardized combinatorial testing methods remain necessary to determine reproducibility and clinical applicability.

### 3.3. Adverse Effects and Safety of Iron Nanoparticles

Despite the promising antimicrobial properties, safety considerations remain critical. Some investigations evaluated potential toxicological aspects of iron nanoparticles [24,27,30,31]. Luppi et al. [30] assessed oxygen uptake rate alterations in microbial systems exposed to nanomaterials, indicating possible metabolic interference at certain concentrations.

Additionally, concerns regarding oxidative stress in non-target cells have been raised due to ROS generation associated with iron-based nanoparticles [15]. Excessive ROS production may induce lipid peroxidation, DNA damage, and inflammatory responses in mammalian cells.

Studies primarily focused on physicochemical characterization, such as El-Bassuony and El-Bassuony [25], did not provide detailed microbiological toxicity parameters, highlighting the need for comprehensive biological safety assessment before application.

It was observed that IONP *Cinnamomum verum* coatings on gastrostomy tubes reduced bacterial biofilms while maintaining, and even enhancing, endothelial cell viability and normal morphology over several days, suggesting a promising safety profile under controlled conditions [16].

Overall, although IONPs demonstrate relevant antibacterial and antibiofilm potential, particularly in combination with antibiotics, further in vivo studies and standardized toxicological evaluations are necessary to establish a safe therapeutic window.

### 3.4. Comparative Analysis of Nanoparticle Characteristics and Biological Effects

Beyond the individual results, a comparative analysis of the included studies reveals correlations between key nanoparticle characteristics and their antibacterial and antibiofilm activity. The synthesis route appears to critically influence efficacy. Iron nanoparticles from biological synthesis, such as those produced using *Streptomyces* sp. or bacterial biomass [20,23,29], frequently exhibited relevant antibacterial effects at lower concentrations when compared with some chemically synthesized formulations, possibly due to the presence of residual organic compound agents that improve interaction with bacterial cells.

Targeted designs further amplified these effects; for instance, mannoside-tagged SPIONs loaded with ciprofloxacin markedly enhanced killing of quinolone-resistant *E. coli* and reduced the ciprofloxacin dose required for biofilm inhibition by up to 16-fold [24].

Consistent with this, antibiotic-loaded Fe_3_O_4_ systems such as the amoxicillin–IONP formulation described by Grumezescu et al. [17] and the ciprofloxacin-loaded SPIONs developed by Atac et al. [24] illustrate how surface functionalization and drug conjugation can markedly shift the activity profile of iron oxide nanoparticles, reducing the doses of conventional antibiotics required to inhibit both Gram-positive and Gram-negative pathogens.

Particle size and surface functionalization emerge as important modulators of activity. Although not all studies reported detailed size distributions, those that provided such information generally associated smaller nanoparticles, as well as particles functionalized with molecules such as amino benzoic acid, gallic acid, or antibiotics [21,27,29], with antibacterial and antibiofilm effects. This is consistent with the notion that reduced sizes increase specific surface area, facilitate membrane contact, and may promote reactive oxygen species (ROS) generation. In contrast, formulations with limited physicochemical characterization or potential aggregation tended to require higher concentrations (in the milligram per milliliter range) to achieve similar inhibitory effects [15,27].

Experimental conditions and endpoints also contribute to variability. Most studies focused on planktonic bacteria and minimum inhibitory concentration (MIC) values, whereas only a subset systematically evaluated biofilm formation or preformed biofilms [18,21,25]. As a result, direct quantitative comparison of MICs is hampered not only by heterogeneous units (µg/mL, mg/mL, mM) but also by differences in strains, inoculum size, and assay protocols. In this context, our synthesis emphasizes qualitative trends rather than strict numerical ranking: overall, iron oxide nanoparticles tend to be more effective against Gram-positive species such as *S. aureus* and *Bacillus* spp. at lower concentrations, whereas higher doses are often required to inhibit Gram-negative bacteria such as *P. aeruginosa* and *K. pneumoniae* [15,20,23,26,29].

Green-synthesized IONPs exhibited measurable antibacterial activity, but silver and zinc oxide nanoparticles obtained from the same plant extract achieved larger inhibition zones against *S. aureus*, *E. faecalis*, *E. coli*, *P. aeruginosa* and *Salmonella* spp., illustrating the more moderate intrinsic antibacterial profile of iron oxide compared with other metal-based systems [32].

Finally, synergistic combinations with antibiotics systematically shifted the activity profile, reducing the concentrations required for bacterial inhibition and biofilm disruption [19,21,23,27]. These findings suggest that, instead of being used as standalone agents, iron oxide nanoparticles may be more rationally positioned as adjuvants that potentiate conventional antibiotics, particularly against multidrug-resistant and biofilm-forming pathogens.

### 3.5. Final Considerations

Iron oxide nanoparticles (IONPs), within the context of nanotechnology, emerge as an innovative and promising strategy in medical research aimed at combating bacterial multidrug resistance. Based on the studies identified and critically analyzed in this review, the guiding question was consistently addressed: IONPs exhibit both bactericidal and bacteriostatic effects, depending on concentration, formulation, and microbial susceptibility.

The collected evidence demonstrates that IONPs possess inhibitory activity against a wide range of Gram-positive and Gram-negative bacteria, in addition to showing relevant antibiofilm potential. These properties reinforce their possible role in the development of alternative antimicrobial strategies, particularly in the face of increasing antimicrobial resistance. Moreover, the demonstrated capacity for association with conventional antibiotics highlights their versatility and potential application as adjuvant agents, capable of enhancing drug efficacy and possibly reducing required therapeutic doses.

However, the antimicrobial effects of IONPs are closely linked to concentration-dependent mechanisms. Determining effective yet safe dosages is essential to avoid cytotoxic effects and oxidative damage to host tissues. The variability in experimental methodologies, nanoparticle synthesis protocols, and microbiological assays observed among studies underscores the need for standardized evaluation models.

Furthermore, most investigations remain limited to in vitro analyses. Additional in vivo studies are necessary to evaluate biodistribution, pharmacokinetics, long-term toxicity, and therapeutic safety. Expanding research in these areas will be fundamental for consolidating scientific knowledge and enabling future applications of IONPs as antimicrobial agents.

## 4. Methods

### 4.1. Study Design

This study was conducted as an integrative systematic review aimed at synthesizing experimental evidence regarding the antibacterial and antibiofilm activity of iron oxide nanoparticles (IONPs). The review followed the Preferred Reporting Items for Systematic Reviews and Meta-Analyses (PRISMA) guidelines [33], and the review protocol was retrospectively registered in the Open Science Framework (OSF) under the registration DOI 10.17605/OSF.IO/95KJR. The PRISMA flow diagram was used to document the identification, screening, eligibility, and inclusion stages of the selected studies, and the completed PRISMA 2020 checklist is provided as Supplementary Material (Table S1).

A comprehensive literature search was performed using the following electronic databases: PubMed (National Library of Medicine); Web of Science; BVS (Virtual Health Library); LILACS (Latin American Literature in Health Sciences); Redalyc (Network of Open Access Scientific Journals). The database search was conducted in August 2025 using English-language descriptors selected according to the DeCS (Health Sciences Descriptors) controlled vocabulary.

The following terms were applied—“Iron nanoparticles”, “Iron oxide nanoparticles”, IONPs, “Bacteria”, “Bacterial inhibition”, and “Biofilm inhibition”—and the Boolean operator “AND” and “OR” were used to combine descriptors as follows: “Iron nanoparticles AND bacteria”, “Iron nanoparticles AND bacterial inhibition”, “Iron oxide nanoparticles AND biofilm inhibition”, “Iron oxide nanoparticles” OR IONPs AND bacterial inhibition”, “Iron oxide nanoparticles” OR IONPs AND biofilm inhibition”. The only restriction applied during the search phase was the exclusion of review articles and meta-analyses.

### 4.2. Eligibility Criteria

Studies were considered eligible if they met the following inclusion criteria: Published between 2014 and 2024, original experimental research articles, written in English, investigated iron or iron oxide nanoparticles, evaluated antibacterial and/or antibiofilm activity, and included Gram-positive and/or Gram-negative bacteria. The exclusion criteria were: duplicate articles, conference abstracts without full text, undergraduate final projects, Master’s dissertations, PhD theses, and studies not evaluating antimicrobial outcomes.

### 4.3. Study Selection Process

After database searches, all identified records were exported and screened for duplicates. Two stages of screening were performed: title and abstract screening, and full-text assessment for eligibility. Studies not meeting the inclusion criteria were excluded.

After full-text analysis, relevant data were extracted, including type of iron nanoparticle, target microorganism, experimental model (in vitro/in vivo), antibacterial outcomes, antibiofilm activity, synergistic effects with antibiotics, and reported adverse or cytotoxic effects.

The selected studies were categorized into three thematic groups: use of iron nanoparticles as antibacterial agents, evaluation of iron nanoparticles in synergy with antibiotics, adverse effects and safety of iron nanoparticles.

### 4.4. Quality Assessment

The methodological quality of the included studies was independently assessed by two reviewers using the Joanna Briggs Institute (JBI) Critical Appraisal Checklist for quasi-experimental studies. The items evaluated included clarity of the experimental design, presence and adequacy of control groups, completeness of nanoparticle physicochemical characterization, reliability of antimicrobial measurements, and description of statistical analyses. Each item was rated as “yes”, “no”, “unclear”, or “not applicable”, and studies were then classified as having low or moderate overall risk of bias. Disagreements were resolved by consensus. As shown in Table 2, most studies were judged to have low risk of bias for control group use and antimicrobial effects, whereas moderate risk was mainly associated with partial nanoparticle characterization and incomplete reporting of statistical methods.

It should also be noted that the JBI checklist was not specifically developed for nanomaterial research and may not fully capture certain sources of bias that are particular to in vitro studies, such as aggregation behavior or long-term stability. Therefore, our risk-of-bias classification, although systematically applied, should be interpreted with caution and in the context of these methodological limitations.

### 4.5. Use of Generative Artificial Intelligence Tools

A schematic illustration of the proposed antibacterial and antibiofilm mechanisms of iron oxide nanoparticles (Figure 2) was created using BioRender.com. The tool was used exclusively for figure assembly and visualization; no generative artificial intelligence was used for data analysis or for generating or editing the textual content of this manuscript.

## 5. Conclusions

Iron oxide nanoparticles (IONPs) demonstrate significant antibacterial and antibiofilm activity against Gram-positive and Gram-negative bacteria, with effects that may be bacteriostatic or bactericidal depending on concentration, formulation, and microbial susceptibility. The studies included in this review also indicate that nanoparticles obtained by green or biological synthesis, as well as those with smaller sizes or specific surface functionalization, tend to be active at lower concentrations and may exhibit enhanced effects on biofilm formation and stability, particularly when combined with conventional antibiotics. In this context, IONPs emerge not only as potential antimicrobial agents but also as promising adjuvants capable of potentiating the effectiveness of existing antimicrobial therapies, especially in biofilm-associated infections.

However, the evidence remains heterogeneous, with important differences in synthesis routes, physicochemical characterization, planktonic and biofilm models, and outcome reporting (including non-standardized MIC units and incomplete description of biofilm assays), which limits direct comparison between studies including a quantitative synthesis. In addition, cytotoxicity and biocompatibility data are still scarce, and most investigations are restricted to in vitro designs, hindering extrapolation to clinical practice.

By integrating antibacterial and antibiofilm findings with nanoparticle characteristics and synergistic interactions with antibiotics, this review offers a comparative perspective rather than a purely descriptive summary. Future studies should adopt more standardized protocols for nanoparticle characterization and antimicrobial testing, systematically address safety and toxicity, and incorporate in vivo or clinically oriented models. Addressing these gaps will be essential to support the rational and safe application of iron oxide nanoparticles as adjuncts or alternatives in antimicrobial therapy.

## Figures and Tables

**Figure 1:**
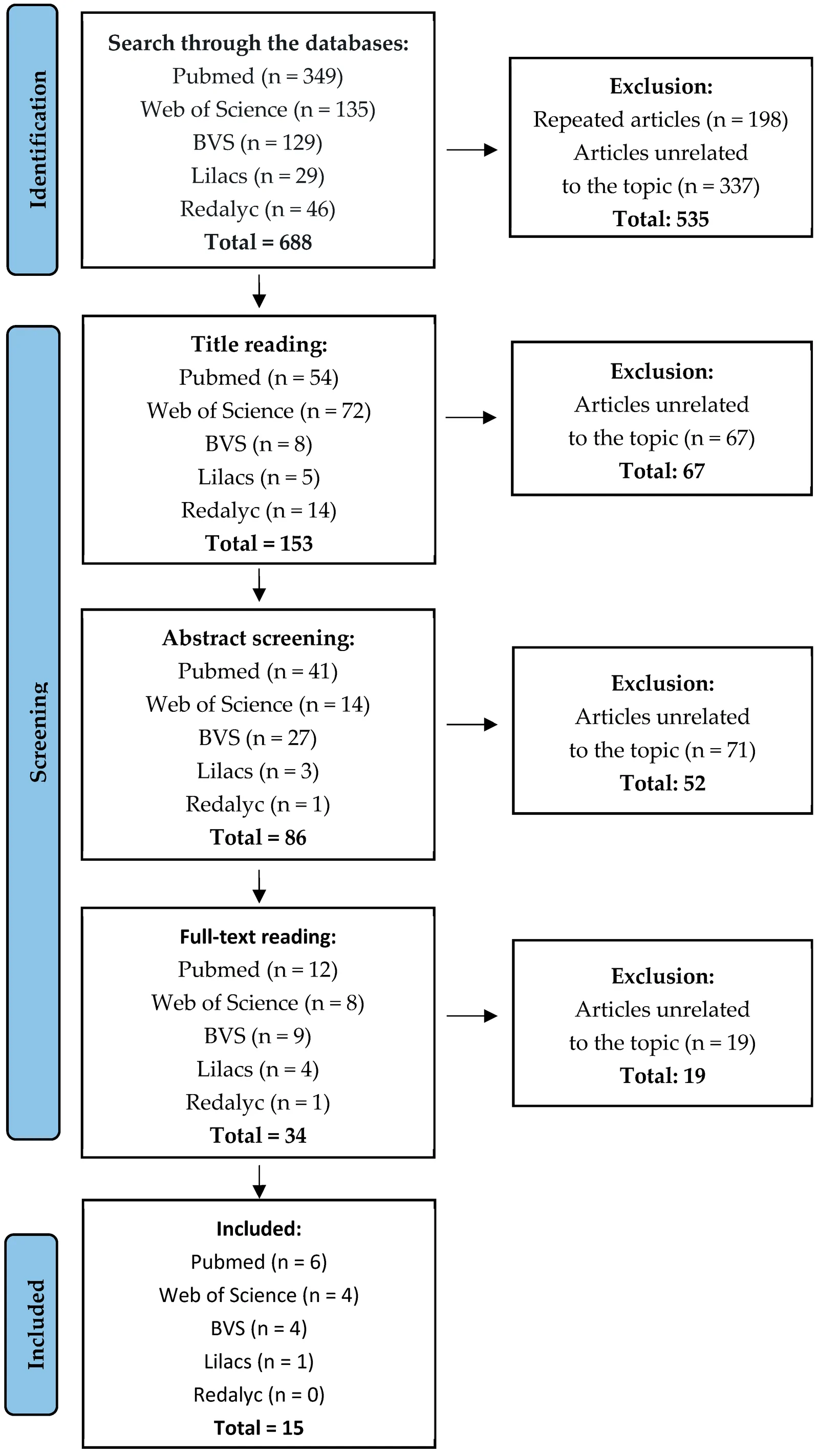
PRISMA flow diagram illustrating the identification, screening, eligibility, and inclusion process of studies retrieved from PubMed, Web of Science, BVS, LILACS, and Redalyc.

**Figure 2:**
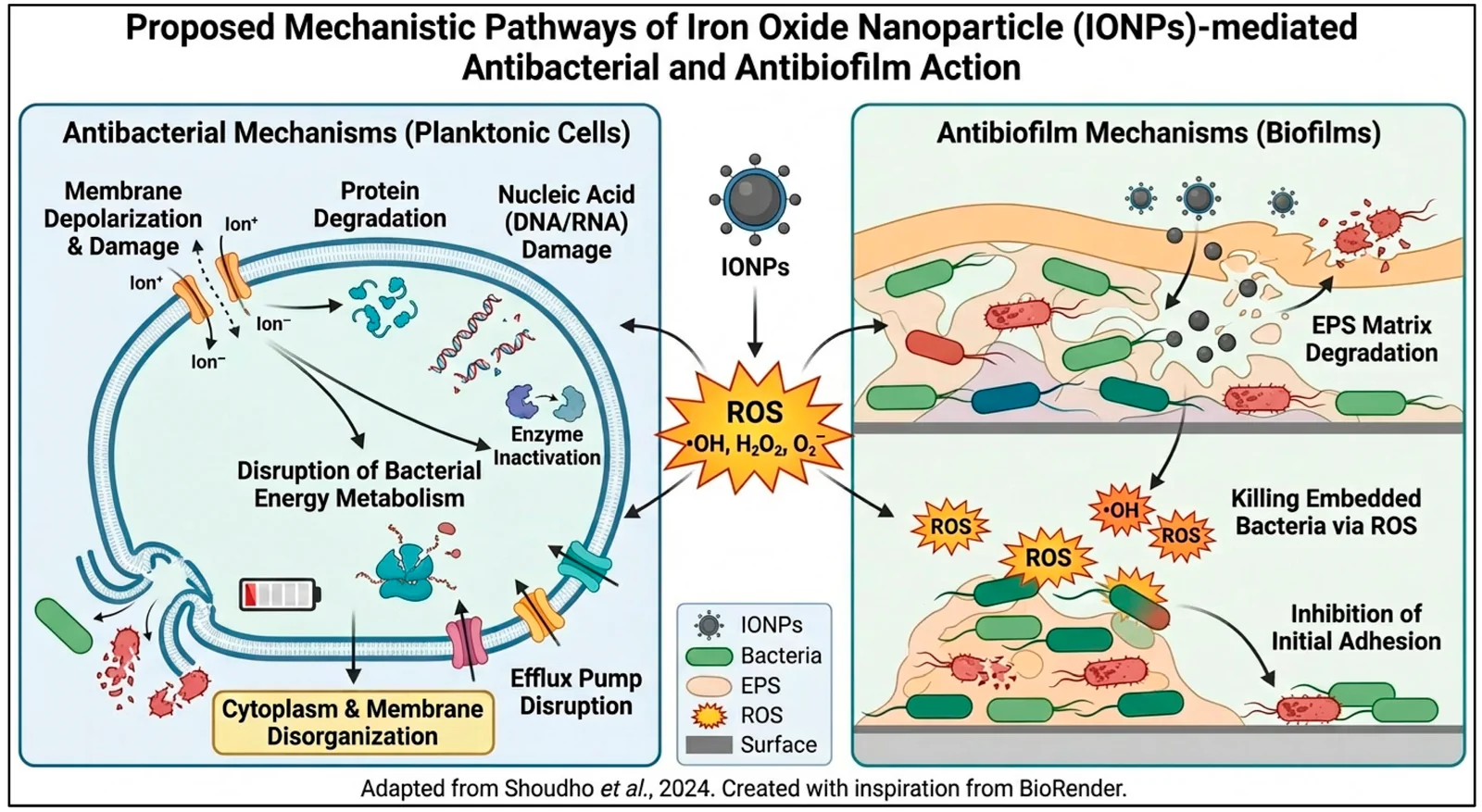
Schematic representation of the proposed antibacterial and antibiofilm mechanisms of iron oxide nanoparticles (IONPs). IONPs induce oxidative stress and promote cell damage through the generation of reactive oxygen species (ROS), leading to membrane depolarization, disruption of the cell envelope, damage to proteins and nucleic acids, enzyme inactivation, interference with efflux pumps, and cytoplasmic disorganization. In addition, IONPs may impair biofilm formation and stability by altering the extracellular matrix and affecting cell–cell interactions. ROS, reactive oxygen species; IONPs, iron oxide nanoparticles. Created with BioRender.com; adapted from Shoudho et al., 2024 [7].

**Table 1:** Descriptive and summarized table of studies included in the bibliographic research.

Study (Year)	Nanoparticle Type	Bacterial Species and Gram (G) Type	Particle Size	Bacterial Inhibition (MIC)	Biofilm Effect	Synergy with Antibiotics
Anghel et al., 2014 [16]	Fe_3_O_4_ NPs incorporated in MAPLE-deposited coatings	*Staphylococcus aureus* (G+), *Escherichia coli* (G−)	9.4 nm	Yes (no MIC reported)	Yes (no MIC reported)	No reported
Grumezescu et al., 2014 [17]	Fe_3_O_4_ NPs biocompatible	*Staphylococcus aureus* (G+), *Escherichia coli* (G−)	10 nm	0.5–2.1 µg/mL	0.5–2.1 µg/mL	Amoxicillin
Gabrielyan et al., 2019 [15]	Fe_3_O_4_ NPs	*Escherichia coli* (G−) and *Enterococcus hirae* (G+)	No reported	250 µg/mL	No reported	No reported
Pham et al., 2019 [18]	FeOOH NPs	*Pseudomonas aeruginosa* (G−)	40 nm	0.6 mM	0.3 mM	No reported
Shahbazi et al., 2019 [19]	Fe_2_O_3_ NPs	*Enterococcus faecalis* (G+)	10–50 nm	39–75 µg/mL	No reported	Ceftazidime; Clindamycin Oxacillin
Rajeswaran et al., 2020 [20]	Fe NPs green-synthesized	*Staphylococcus aureus* (G+), *Bacillus subtilis* (G+), *Klebsiella pneumonia* (G−), *Shigella flexneri* (G−), *Escherichia coli* (G−)	65 to 86 nm	50–75 µg/mL	No reported	No reported
Armijo et al., 2020 [21]	Fe_3_O_4_, Fe_4_N NPs and tobramycin-conjugated	*Pseudomonas aeruginosa* (G−)	15–65 nm	10 µg/mL	17 mg/mL	Tobramycin
Zafar et al., 2022 [22]	Fe NPs	*P. aeruginosa* (G−), *Providencia stuartii* (G−), *E. coli* (G−) and *S. aureus* (G+).	23 nm	25–100 µg/mL	No reported	No reported
Abdulsada et al., 2022 [23]	Fe_3_O_4_-gentamicin NPs	*Staphylococcus epidermidis* (G+), *Proteus mirabilis* (G−) and *Acinetobacter baumannii* (G−)	21–55 nm	25–100 µg/mL	100 µg/mL	Gentamicin
Atac et al., 2023 [24]	Fe_3_O_4_ mannosídeo + ciprofloxacino.	*Escherichia coli* (G−) ciprofloxacin-resistant	4.5–41 nm	52 µg/mL	52 µg/mL	Ciprofloxacin
El-Bassuony et al., 2023 [25]	Ag-Fe_3_O_4_ nanocomposite	*S. aureus* (G+), *S. faecalis* (G+), *B. subtilis* (G+), *E. coli* (G−), *Neisseria gonorrhoeae* (G−), *P. aeruginosa* (G−)	56–71 nm	Yes (MIC no reported)	No reported	No reported
Faisal et al., 2023 [26]	Fe NPs modified with amino benzoic acid	*S. aureus* (G+)	62 nm	30 mg/mL	No reported	No reported
De Sousa et al., 2023 [27]	Fe_3_O_4_ NPs associated with rifampicin	*S. aureus* (G+).	8.3–57 nm	6 mg/mL	No reported	Rifampicin
Fatih et al., 2024 [28]	α-Fe_2_O_3_ NPs (green-synthesized)	*Bacillus* sp. (G+)	29–36 nm	0.6 µg/mL	0.3 µg/mL	No reported
Khaleel et al., 2024 [29]	Fe_3_O_4_ NPs coated with gallic acid	*K. pneumoniae* (G−)	32–61 nm	3 mg/mL	3 mg/mL	No reported

**Table 2:** Methodological quality assessment of included studies according to the Joanna Briggs Institute (JBI) Critical Appraisal Checklist.

Study	Control Group	Nanoparticle Characterization	Outcome Measurement	Statistical Analysis	Overall Risk of Bias
Anghel et al., 2014 [16]	Yes	Yes	Yes	Yes	Low
Grumezescu et al., 2014 [17]	Yes	Yes	Yes	Yes	Low
Gabrielyan et al., 2019 [15]	Yes	Yes	Yes	Yes	Low
Pham et al., 2019 [18]	Yes	Yes	Yes	Yes	Low
Shahbazi et al., 2019 [19]	Yes	Partial	Yes	Partial	Moderate
Rajeswaran et al., 2020 [20]	Yes	Partial	Yes	Yes	Moderate
Armijo et al., 2020 [21]	Yes	Yes	Yes	Yes	Low
Zafar et al., 2022 [22]	Yes	Yes	Yes	Yes	Low
Abdulsada et al., 2022 [23]	Yes	Yes	Yes	Yes	Low
Atac et al., 2023 [24]	Yes	Yes	Yes	Yes	Low
El-Bassuony et al., 2023 [25]	Yes	Yes	Yes	Yes	Low
Faisal et al., 2023 [26]	Yes	Yes	Yes	Yes	Low
De Sousa et al., 2023 [27]	Yes	Yes	Yes	Yes	Low
Fatih et al., 2024 [28]	Yes	Yes	Yes	Yes	Low
Khaleel et al., 2024 [29]	Yes	Partial	Yes	Yes	Moderate

## Data Availability

No new data were created or analyzed in this study. Data sharing is not applicable to this article.

## Supplementary Materials

The following supporting information can be downloaded at: https://www.mdpi.com/article/10.3390/antibiotics15101014/s1. Table S1: PRISMA 2020 Checklist.

## Abbreviations

The following abbreviations are used in this manuscript:

AMR	Antimicrobial resistance
IONPs	Iron oxide nanoparticles
ROS	Reactive oxygen species
MIC	Minimum inhibitory concentration
JBI	Joanna Briggs Institute
PRISMA	Preferred Reporting Items for Systematic Reviews and Meta-Analyses
