# Supplementary material for: Antibacterial and Antibiofilm Activity of Iron Oxide Nanoparticles: An Integrative Systematic Review
Source: Antibiotics (Basel). 2026 Oct 13;15(10):1014. doi: 10.3390/antibiotics15101014 (PMC13700858; doi:10.3390/antibiotics15101014)
Supplement: Supplementary file 1 [file antibiotics-15-01014-s001.zip › antibiotics-4194481-supplementary.pdf]

PRISMA

PRISMA 2020

Checklist Table S1. PRISMA 2020

Checklist for reporting systematic reviews, indicating page numbers and manuscript locations where each item is addressed.

Manuscript: Antibacterial and Antibiofilm Activity of Iron Oxide Nanopar-ticles: An Integrative Systematic Review

Authors: Jessica Alves dos Santos, Adriana Bozzi, Marianna Gabrielle Almeida Galvão, Aline Silva.

Section and Topic	Item #	Checklist item	Location where item is reported
**TITLE**			
Title	1	Identify the report as a systematic review.	**Main title**
**ABSTRACT**			
Abstract	2	See the PRISMA 2020 for Abstracts checklist.	**Abstract Section,** Contains: Background, Methods, Results, Conclusions.
**INTRODUCTION**			
Rationale	3	Describe the rationale for the review in the context of existing knowledge.	**Introduction, paragraphs 1-3:** Justification: Antimicrobial resistance, therapeutic limitations, potential of nanoparticles.
Objectives	4	Provide an explicit statement of the objective(s) or question(s) the review addresses.	**End of section 1,** last paragraph of the Introduction.
**METHODS**			
Eligibility criteria	5	Specify the inclusion and exclusion criteria for the review and how studies were grouped for the syntheses.	**Section 4.2 and 4.3:** Eligibility criteria inclusion and exclusion criteria described.
Information sources	6	Specify all databases, registers, websites, organisations, reference lists and other sources searched or consulted to identify studies. Specify the date when each source was last searched or consulted.	**Section 4.1:** Earch strategy, databases: PubMed; Web of Science; BVS; LILACS; Redalyc. The search was conducted in August 2025.
Search strategy	7	Present the full search strategies for all databases, registers and websites, including any filters and limits used.	**Section 4.1:** The following terms were applied: “Iron nanoparticles”, “Iron oxide nanoparticles”, IONPs, “Bacteria”, “Bacterial inhibition”, “Biofilm inhibition” and the Boolean operator “AND” and “OR” were used to combine descriptors as follows: “Iron nanoparticles AND bacteria”, “Iron nanoparticles AND bacterial inhibition”, “Iron oxide nanoparticles AND biofilm inhibition”, “Iron oxide nanoparticles” OR IONPs AND bacterial inhibition”, “Iron oxide nanoparticles” OR IONPs AND biofilm inhibition”,. English-language descriptors selected according to the DeCS (Health Sciences Descriptors) controlled vocabulary.The terms were applied: “Iron nanoparticles”, “Bacteria”, “Bacterial inhi-bition”, and the Boolean operator “AND” used to combine descriptors.
Selection process	8	Specify the methods used to decide whether a study met the inclusion criteria of the review, including how many reviewers screened each record and each report retrieved, whether they worked independently, and if applicable, details of automation tools used in the process.	**Section 4.3 and 4.4:** To study selection process all identified records were exported and screened for du-plicates. Two stages of screening were performed: Title and abstract screening, full-text assessment for eligibility. The included studies was independently assessed by two reviewers using the Joanna Briggs Institute (JBI) Critical Appraisal Checklist for quasi experimental studies. Disagreements were resolved by consensus.
Data collection process	9	Specify the methods used to collect data from reports, including how many reviewers collected data from each report, whether they worked independently, any	**Section 4.4:** Two reviewers independently extracted data using a standardized form. Disagreements were resolved by consensus. No automation tools were used, and study authors were not contacted.

Checklist Table S1. PRISMA 2020

Checklist for reporting systematic reviews, indicating page numbers and manuscript locations where each item is addressed.

Manuscript: Antibacterial and Antibiofilm Activity of Iron Oxide Nanopar-ticles: An Integrative Systematic Review

Authors: Jessica Alves dos Santos, Adriana Bozzi, Marianna Gabrielle Almeida Galvão, Aline Silva.

Section and Topic	Item #	Checklist item	Location where item is reported
		processes for obtaining or confirming data from study investigators, and if applicable, details of automation tools used in the process.	
Data items	10a	List and define all outcomes for which data were sought. Specify whether all results that were compatible with each outcome domain in each study were sought (e.g. for all measures, time points, analyses), and if not, the methods used to decide which results to collect.	**Section 4.3:** The primary outcome was antimicrobial activity (e.g., MIC, MBC, biofilm inhibition). Secondary outcomes included mechanisms of action, synergism with antibiotics, nanoparticle characteristics, and toxicity.
	10b	List and define all other variables for which data were sought (e.g. participant and intervention characteristics, funding sources). Describe any assumptions made about any missing or unclear information.	**Section 4.4:** Other variables included nanoparticle characteristics, bacterial species, experimental model, antimicrobial assays, antibiotic association, toxicity data. Only explicitly available data were extracted, with no assumptions made for missing information.
Study risk of bias assessment	11	Specify the methods used to assess risk of bias in the included studies, including details of the tool(s) used, how many reviewers assessed each study and whether they worked independently, and if applicable, details of automation tools used in the process.	**Section 4.4 and Table 2**: Two reviewers independently assessed methodological quality using the JBI Critical Appraisal Checklist for Quasi-Experimental Studies**,** with disagreements resolved by consensus. No automation tools were used.
Effect measures	12	Specify for each outcome the effect measure(s) (e.g. risk ratio, mean difference) used in the synthesis or presentation of results.	**Section 4.3 and 3 (Results):** No meta-analysis was conducted; outcomes (e.g., MIC, MBC, inhibition zones, biofilm inhibition) were descriptively reported as presented in the included studies.
Synthesis methods	13a	Describe the processes used to decide which studies were eligible for each synthesis (e.g. tabulating the study intervention characteristics and comparing against the planned groups for each synthesis (item #5).	**Sections 4.2, 4.3, and 3 (Results)**: Studies were selected based on predefined inclusion criteria and grouped according to nanoparticle characteristics and reported antimicrobial outcomes for narrative synthesis.
	13b	Describe any methods required to prepare the data for presentation or synthesis, such as handling of missing summary statistics, or data conversions.	**Sections 4.3:** No quantitative data conversions or statistical transformations were required, as results were synthesized narratively.
	13c	Describe any methods used to tabulate or visually display results of individual studies and syntheses.	**Section 4.3, Figure 1, Tables 1–2 and Results:** Results were presented in structured tables (Tables 1 and 2) summarizing study characteristics and methodological quality, and visually displayed using the PRISMA flow diagram (Figure 1). Synthesis was narrative.
	13d	Describe any methods used to synthesize results and provide a rationale for the choice(s). If meta-analysis was performed, describe the model(s), method(s) to identify the presence and extent of statistical heterogeneity, and software package(s) used.	**Section 4.3 and Section 2 and 3:** Results were synthesized narratively due to methodological heterogeneity. No meta-analysis, statistical models, or software were used.

PRISMA

PRISMA 2020

Checklist Table S1. PRISMA 2020

Checklist for reporting systematic reviews, indicating page numbers and manuscript locations where each item is addressed.

Manuscript: Antibacterial and Antibiofilm Activity of Iron Oxide Nanopar-ticles: An Integrative Systematic Review

Authors: Jessica Alves dos Santos, Adriana Bozzi, Marianna Gabrielle Almeida Galvão, Aline Silva.

Section and Topic	Item #	Checklist item	Location where item is reported
	13e	Describe any methods used to explore possible causes of heterogeneity among study results (e.g. subgroup analysis, meta-regression).	**Section 4.3 and Results:** No statistical exploration of heterogeneity was performed. Variability was addressed descriptively through thematic grouping of studies.
	13f	Describe any sensitivity analyses conducted to assess robustness of the synthesized results.	**Section 4.3, Results and Discussion:** No sensitivity analyses were conducted, as no meta-analysis was performed. The synthesis was narrative and based on thematic grouping of the included studies.
Reporting bias assessment	14	Describe any methods used to assess risk of bias due to missing results in a synthesis (arising from reporting biases).	**Section 4.4 and Table 2.** Risk of bias was assessed using the JBI Critical Appraisal Checklist for quasi-experimental studies. Two independent reviewers performed the evaluation, and studies were classified as low or moderate risk of bias.
Certainty assessment	15	Describe any methods used to assess certainty (or confidence) in the body of evidence for an outcome.	**Section 4.4:** No formal method was used to assess certainty of evidence. Only individual study quality was evaluated using the JBI tool.
**RESULTS**			
Study selection	16a	Describe the results of the search and selection process, from the number of records identified in the search to the number of studies included in the review, ideally using a flow diagram.	**Section 2, 3 and illustrated in Figure 1 (PRISMA flow diagram):** Records were identified, screened, and assessed for eligibility, resulting in 15 studies included in the qualitative synthesis.
	16b	Cite studies that might appear to meet the inclusion criteria, but which were excluded, and explain why they were excluded.	**Section 2, 3 and Figure 1 (PRISMA flow diagram):** Studies that initially appeared to meet the inclusion criteria but were excluded after full-text assessment,<br>the main reasons for exclusion included: lack of specific evaluation of antimicrobial activity of iron-based nanoparticles, studies not involving bacterial models and insufficient methodological detail.
Study characteristics	17	Cite each included study and present its characteristics.	**Section 2, 3.2 and detailed in Table 1:** The review includes 15 studies: Gabrielyan et al., 2019; Pham et al., 2019; Shahbazi et al., 2019; Rajeswaran et al., 2020; Armijo et al., 2020; Zafar et al., 2022; Abdulsada et al., 2022; El-Bassuony et al., 2023; Faisal et al., 2023; De Sousa et al., 2023; Fatih et al., 2024; Khaleel et al., 2024; Anghel et al, 2014; Grumezescu et al, 2014; Atac et al, 2023.<br><br>For each study, the following characteristics are reported (Table 1): Nanoparticle type, Bacterial Species and Gram (G) type, Particle size, Bacterial inhibition (MIC), Biofilm effect, Synergy with Antibiotics.
Risk of bias in studies	18	Present assessments of risk of bias for each included study.	**Section 4.4 and Table 2:** Risk of bias was assessed independently by two reviewers using the JBI checklist, and each study was classified as low or moderate risk. Individual results are shown in Table 2.

PRISMA

PRISMA 2020

Checklist Table S1. PRISMA 2020

Checklist for reporting systematic reviews, indicating page numbers and manuscript locations where each item is addressed.

Manuscript: Antibacterial and Antibiofilm Activity of Iron Oxide Nanopar-ticles: An Integrative Systematic Review

Authors: Jessica Alves dos Santos, Adriana Bozzi, Marianna Gabrielle Almeida Galvão, Aline Silva.

Section and Topic	Item #	Checklist item	Location where item is reported
Results of individual studies	19	For all outcomes, present, for each study: (a) summary statistics for each group (where appropriate) and (b) an effect estimate and its precision (e.g. confidence/credible interval), ideally using structured tables or plots.	**Section 2, 3 and Table 1:** Individual study results are presented descriptively in Section 3 (Results) and summarized in Table 1. Reported outcomes include antimicrobial measures such as MIC, inhibition zones, and biofilm reduction. No pooled effect estimates or confidence intervals were calculated, as no meta-analysis was performed.
Results of syntheses	20a	For each synthesis, briefly summarise the characteristics and risk of bias among contributing studies.	**Section 2 and 3, Table 1 and 4.4:** For each synthesis, study characteristics summarized in Section 3 (Results) and detailed in Table 1, while methodological quality and risk of bias are presented in "Quality Assessment" and summarized in Table 2.
	20b	Present results of all statistical syntheses conducted. If meta-analysis was done, present for each the summary estimate and its precision (e.g. confidence/credible interval) and measures of statistical heterogeneity. If comparing groups, describe the direction of the effect.	**Section 2 and 3:** No statistical syntheses were conducted, as no meta-analysis was performed due to methodological heterogeneity among studies. Results were synthesized narratively, describing the direction of antimicrobial effects across studies.
	20c	Present results of all investigations of possible causes of heterogeneity among study results.	**Section 2 and 3:** No formal heterogeneity analyses were performed. Variability was addressed descriptively through thematic grouping.
	20d	Present results of all sensitivity analyses conducted to assess the robustness of the synthesized results.	No sensitivity analyses were conducted, as no meta-analysis was performed.
Reporting biases	21	Present assessments of risk of bias due to missing results (arising from reporting biases) for each synthesis assessed.	**Section 4,4 and Table 2:** No specific assessment of risk of bias due to missing results was conducted, as no meta-analysis or quantitative synthesis was performed. However, overall methodological quality of included studies was evaluated using the JBI Critical Appraisal Checklist.
Certainty of evidence	22	Present assessments of certainty (or confidence) in the body of evidence for each outcome assessed.	No formal certainty assessment was performed; confidence was inferred from JBI quality appraisal and consistency of findings.
**DISCUSSION**			
Discussion	23a	Provide a general interpretation of the results in the context of other evidence.	**Section 2 and 3:** Iron-based nanoparticles demonstrate promising antibacterial and antibiofilm activity, particularly as adjuncts to antibiotics against bacterial strains.
	23b	Discuss any limitations of the evidence included in the review.	**Section 3:** The evidence is limited by methodological heterogeneity, variability in nanoparticle characteristics, and the predominance of in vitro studies, restricting comparability and generalizability.
	23c	Discuss any limitations of the review processes used.	**Section 5:** The review process was limited by the selection of specific databases, predefined search terms, and the defined publication period, which may have led to omission of relevant studies.
	23d	Discuss implications of the results for practice, policy, and	**Section 5 and 6:** Findings support iron-based nanoparticles as potential

PRISMA

PRISMA 2020

Checklist Table S1. PRISMA 2020

Checklist for reporting systematic reviews, indicating page numbers and manuscript locations where each item is addressed.

Manuscript: Antibacterial and Antibiofilm Activity of Iron Oxide Nanopar-ticles: An Integrative Systematic Review

Authors: Jessica Alves dos Santos, Adriana Bozzi, Marianna Gabrielle Almeida Galvão, Aline Silva.

Section and Topic	Item #	Checklist item	Location where item is reported
		future research.	adjuncts to antibiotics, but clinical application requires standardization, safety evaluation, and further in vivo research.
**OTHER INFORMATION**			
Registration and protocol	24a	Provide registration information for the review, including register name and registration number, or state that the review was not registered.	This review was registered in database OSF, DOI 10.17605/OSF.IO/95KJR.
	24b	Indicate where the review protocol can be accessed, or state that a protocol was not prepared.	No review protocol was prepared for this study.
	24c	Describe and explain any amendments to information provided at registration or in the protocol.	This review was registered in database OSF, DOI 10.17605/OSF.IO/95KJR.
Support	25	Describe sources of financial or non-financial support for the review, and the role of the funders or sponsors in the review.	This research received financial support solely in the form of a scholarship awarded to J.A.S. The funding source had no role in the design, analysis, or reporting of the review.
Competing interests	26	Declare any competing interests of review authors.	The authors declare no conflict of interest.
Availability of data, code and other materials	27	Report which of the following are publicly available and where they can be found: template data collection forms; data extracted from included studies; data used for all analyses; analytic code; any other materials used in the review.	All relevant data are available within the article. No additional datasets or analytic code are publicly available.

From: Page MJ, McKenzie JE, Bossuyt PM, Boutron I, Hoffmann TC, Mulrow CD, et al. The PRISMA 2020 statement: an updated guideline for reporting systematic reviews. BMJ 2021;372:n71. doi: 10.1136/bmj.n71. This work is licensed under CC BY 4.0. To view a copy of this license, visit https://creativecommons.org/licenses/by/4.0/
